# Feasibility and strategy analysis of radiotherapy consolidation following immunotherapy for stage IV esophageal squamous cell carcinoma

**DOI:** 10.3389/fonc.2026.1880587

**Published:** 2026-06-19

**Authors:** Hao Su, Xinyu Zhao, Xiaoyu Qin, Xu Zhou, Mianli Li, Lijun Tian

**Affiliations:** Department of Oncology, Binzhou Medical University Hospital, Binzhou, Shandong, China

**Keywords:** consolidation therapy, immunotherapy, radiotherapy, stage IV esophageal cancer, upper gastrointestinal cancers

## Abstract

Esophageal cancer is one of the most harmful upper gastrointestinal cancers, particularly in its advanced stages. Squamous cell carcinoma accounts for approximately 85% of all esophageal cancer cases among Chinese patients and is the main pathological type. Although first-line immunotherapy combined with chemotherapy has improved survival rates for stage IV esophageal squamous cell carcinoma (ESCC), some patients still experience suboptimal local control due to resistance to the treatment regimen. In patients with stage IV ESCC, disease progression at the primary site typically represents the main pattern of overall disease progression. Preclinical studies have clearly demonstrated a synergistic mechanism between radiotherapy and immunotherapy, and clinical evidence strongly suggests that the addition of consolidation radiotherapy can effectively enhance local lesion control and significantly prolong survival. Therefore, this review aims to explore the mechanisms of action of consolidation radiotherapy following first-line immunotherapy combined with chemotherapy in patients with stage IV ESCC, and to further analyze its feasibility and implementation strategies.

## 1 Introduction

Esophageal malignant tumors rank among the most prevalent digestive tract malignancies globally, exhibiting high incidence and mortality rates (1, 2). According to 2022 GLOBOCAN data, esophageal cancer accounted for approximately 511,000 new cases and 445,000 deaths worldwide, ranking as the 11th most common cancer and the 7th leading cause of cancer death globally. Among the cancer burden in China, esophageal cancer ranks fifth in mortality. During the same period, China recorded approximately 224,000 new esophageal cancer cases and 188,000 deaths, accounting for about 50% of global cases and deaths. Compared to 2020 (approximately 600,000 new cases and 540,000 deaths), the absolute number of esophageal cancer cases worldwide slightly decreased in 2022. However, China still accounted for over half of all global cases (3, 4). It is projected that by 2040, there will be 957,000 new cases of esophageal cancer worldwide (5). Among Chinese populations, 85% of patients present with squamous cell carcinoma, while only approximately 15% have adenocarcinoma. Due to the lack of specific early-stage symptoms, most patients are diagnosed at an advanced stage (6). Approximately 40% of patients have organ metastasis at first diagnosis, and the 5-year overall survival (OS) rate for stage IV esophageal cancer cases is below 5% (7).

According to the 9th Edition American Joint Committee on Cancer (AJCC) Staging Guidelines, stage IV esophageal cancer can be further subdivided into stage IVa and stage IVb: IVa stage is defined as tumors that have invaded adjacent key structures or metastasized to multiple regional lymph nodes (such as lower thoracic tumors metastasizing to abdominal lymph nodes and upper thoracic tumors metastasizing to cervical lymph nodes) without distant organ metastasis; IVb stage is defined as a tumor that has spread beyond the local boundary and undergone distant metastasis (8–10). In the era before immunotherapy, patients with stage IVa disease typically underwent radical concurrent chemoradiotherapy or chemotherapy followed by radiotherapy. For stage IVb patients, systemic chemotherapy served as the primary treatment, with radiotherapy employed as palliative therapy to further control localized lesions (11). For both stage IVa and stage IVb patients, radiotherapy and chemotherapy are effective treatment options. For example, a clinical trial conducted by Chen et al. (12) evaluated the efficacy of a chemotherapy combined with radiotherapy regimen in 436 patients with esophageal squamous cell carcinoma (ESCC), including 52 stage IVa patients and 42 stage IVb patients with supraclavicular lymph node metastasis. All patients received radiotherapy with a total dose of 61.2 Gy. The final results showed that the median overall survival (mOS) reached 47.6 months after adding radiotherapy. This represents a significant improvement compared to historical data from previous studies in stage IV esophageal cancer patients receiving either chemotherapy alone (approximately 7–11 months) or radiotherapy alone (approximately 9 months) (13–16).

The advent of immunotherapy has opened new avenues for cancer treatment. Since immune checkpoint inhibitors (ICIs) were first approved for use in melanoma in 2011, they have been applied to over 20 diseases. In recent years, immunotherapy has significantly improved the survival outcomes of esophageal cancer patients and demonstrated great potential in the treatment of other thoracic tumors (17–19).

Immunotherapy has extended survival in patients with stage IV esophageal cancer. In a phase III clinical trial (KEYNOTE-590) evaluating pembrolizumab or placebo combined with first-line chemotherapy for advanced esophageal cancer, the study enrolled 749 patients, of whom 683 (91.2%) had stage IV disease. Results demonstrated that patients with PD-L1 expression ≥10% in the pembrolizumab plus chemotherapy group achieved significantly improved mOS compared to the placebo plus chemotherapy group (mOS: 13.9 months vs 8.8 months) (20). In another Phase III clinical trial (ESCORT-1ST) (21) involving 596 patients with stage IV disease, combination therapy with Camrelizumab and chemotherapy demonstrated superior mOS (15.3 months vs 12.0 months) and median progression-free survival (mPFS) (6.9 months vs 5.6 months) compared to chemotherapy alone in patients with stage IV ESCC. The above studies have demonstrated that immunotherapy combined with chemotherapy significantly prolongs survival in patients with stage IV esophageal cancer compared to chemotherapy alone.

Although the efficacy of immunotherapy combined with chemotherapy for stage IV esophageal cancer has been confirmed, the issues associated with this treatment approach cannot be overlooked. For example, in a retrospective study (22) comparing recurrence rates after different neoadjuvant immunotherapy-combined-chemotherapy (NICT) regimens, 70 patients who underwent NICT and achieved pathological complete response (pCR) were enrolled. The recurrence rate was 7.14%, with 80.00% of patients experiencing local recurrence. This suggests that patients receiving immunotherapy combined with chemotherapy may still face challenges with suboptimal local control.

To address this issue, radiotherapy may play a crucial role as a localized treatment modality. Under the current immunotherapy-combined-chemotherapy regimen, no large-scale clinical studies have demonstrated the efficacy of radiotherapy in achieving local control for stage IV esophageal cancer. Therefore, this paper aims to discuss the feasibility of adjuvant radiotherapy following first-line chemotherapy combined with immunotherapy for stage IV esophageal cancer patients, as well as the optimal timing for incorporating radiotherapy and the necessary adjustments to radiation dose and target fields.

## 2 Synergistic effects of immunotherapy and radiotherapy

Radiotherapy not only induces tumor shrinkage at the irradiated site but also possesses a “distant effect.” This refers to the phenomenon where tumor shrinkage at the irradiated site simultaneously induces tumor shrinkage at metastatic sites distant from the irradiation field (23, 24). Furthermore, reports indicate that the combined use of immunotherapy and radiotherapy can enhance the therapeutic efficacy of distant effects (25, 26). Additionally, radiotherapy can induce immune responses at sites beyond the irradiated area, thereby amplifying the therapeutic benefits of immunotherapy (27–29). The specific mechanism underlying this effect remains unclear. Research has demonstrated that radiotherapy can increase systemic blood flow, enabling T lymphocytes to circulate throughout the body and infiltrate tumor tissues. This process subsequently leads to a reduction or disappearance of lymph nodes or tumors at metastatic sites (30).

In addition, radiotherapy can transform locally malignant tumors from “cold tumors” to “hot tumors.” This alteration upregulates PD-L1 expression on tumor cells, thereby enhancing immunotherapy efficacy (31). Furthermore, evidence indicates that radiotherapy can enhance the expression of surface markers on immunogenic cells (ICAM-1, MHC-I, Fas), enabling the immune system to rapidly respond to abnormal cellular changes and preventing immune escape by tumor cells (32, 33). Radiotherapy also affects the body’s immune system. Studies indicate that radiation treatment can trigger the release of tumor-associated antigens (TAAs) and endogenous danger signals—damage-associated molecular patterns (DAMPs). This leads to the recognition of these antigens by dendritic cells (DCs), which then present them to T lymphocytes. Consequently, tumor cells are recruited to the site, and local immune regulation is induced (34, 35). Moreover, radiotherapy can induce DNA damage by generating reactive oxygen species (ROS), which can trigger immunogenic cell death (ICD) in tumor cells, thereby enhancing the immune system’s recognition of tumor cells (36). Research also indicates that radiation-induced damage to cytoplasmic DNA can activate the cGAS-STING pathway, inducing the secretion of interferon-β (IFN-β) to further kill tumor cells (37).

Overall, the combination of immunotherapy and radiotherapy is supported by data in terms of its mechanisms, and both their individual and synergistic antitumor effects are highly promising. However, further research is still needed to clarify the specific mechanisms involved.

## 3 The feasibility of adding consolidation radiotherapy

### 3.1 The feasibility of adding consolidation radiotherapy for IVa patients

The most prominent symptoms of cancer IVa stage patients with local invasion as the main feature, as well as local IVb stage patients with specific lymph node metastasis, are local manifestations such as swallowing difficulties, local pain, hoarseness, and cough (38). As radiotherapy is the primary modality for local control, what was its role in the pre-immunotherapy era? For example, in a single-center prospective cohort study (enrolling 102 esophageal cancer patients, including 45 stage IVa and 57 stage IVb patients), the combination chemotherapy plus radiotherapy group demonstrated a significantly higher 1-year dysphagia remission rate compared to the chemotherapy-only group (68% vs 42%, respectively) (39). In addition, similar findings can also be observed in other clinical studies of cancer at different stages. Palliative radiotherapy is usually effective in alleviating local symptoms of swallowing difficulties. For example, Akl et al. (40) recruited 25 patients who presented with swallowing difficulties, and compared to the chemotherapy only group, the addition of palliative radiotherapy showed a higher remission rate (approximately 72% vs. 42%). It is evident that radiotherapy, as the primary means of local control, demonstrates an excellent local symptom relief rate when combined with chemotherapy in the treatment of stage IVa patients.

### 3.2 The feasibility of adding consolidation radiotherapy for IVb patients

For stage IVb patients, in whom distant metastasis has already occurred, the primary goal is to reduce the overall tumor burden. Radiotherapy plays a pivotal role in symptom relief. In a retrospective study by Lyu et al. (16) evaluating radiotherapy efficacy (encompassing 141 stage IVb patients), radiotherapy was administered concurrently with chemotherapy. Fifty-five patients received concurrent chemoradiotherapy, while 86 patients received chemotherapy alone. The final results demonstrated that the concurrent chemoradiotherapy group achieved significantly superior outcomes compared to the chemotherapy-only group in terms of objective response rate (ORR), 1-year progression-free survival (PFS), and 3-year OS (74.5% vs 45.3%, 10.7% vs 4.7%, and 29.8% vs 14.9%, respectively). Additionally, in a single-arm retrospective study by Ikeda et al. (41) evaluating concurrent chemoradiotherapy (encompassing 40 patients, all stage IVb with dysphagia), radiotherapy served as a means to alleviate dysphagia symptoms originating from the primary lesion. The final results demonstrated 95% control of primary lesions, with 30% of patients achieving complete remission. The ORR of 55% surpassed the 45.3% ORR observed in the aforementioned chemotherapy-only group. Consequently, the addition of radiotherapy not only met the need for further tumor burden reduction in stage IVb esophageal cancer patients but also resulted in significant shrinkage of primary tumors.

### 3.3 The feasibility of combining immunotherapy with consolidation radiotherapy

In the era of immunotherapy, is the addition of radiotherapy feasible? A single-arm, phase II EC-CRT-001 clinical trial (42) targeting patients with unresectable esophageal cancer enrolled 42 patients in good general condition (Eastern Cooperative Oncology Group (ECOG) 0-2, well-preserved organ function, no severe autoimmune diseases). Among them were 16 patients with stage IVa esophageal cancer (38%) and 26 patients with stage I-III esophageal cancer (62%). All patients received toripalimab combined with radical concurrent chemoradiotherapy. In this trial, 21 patients completed the planned 17 cycles of immunotherapy, with radiotherapy (50.4 Gy/28 fractions) initiated concurrently on day 1 of immunotherapy. The clinical trial ultimately demonstrated higher rates of pCR and mPFS compared to 84 patients at the same center who received only radical concurrent chemoradiotherapy (62% vs 42% and 12.2 months vs 9.0 months, respectively). EC-CRT-001 suggests that for patients in generally good condition, adding radiotherapy during immunotherapy may effectively prolong the rate of pCR. In a multicenter retrospective study by Hu et al. (43) examining radiotherapy following initial chemotherapy combined with immunotherapy in esophageal cancer patients, researchers enrolled 72 patients with locally advanced or distant metastatic disease. This cohort included 20 patients with oligometastatic disease (≤5 lesions, ≤3 organs) and 10 patients with polymetastatic disease. The study ultimately found that approximately 60% of patients achieved complete remission (CR) or partial remission (PR) with first-line immunotherapy combined with chemotherapy. Following four cycles of immunotherapy-chemotherapy combination therapy, patients received radiotherapy (33.3% underwent primary site irradiation only, while 66.7% received radiation to both primary and metastatic sites). The study demonstrated a mPFS of 13.5 months and a mOS of 31.8 months, with particularly favorable outcomes observed in patients with oligometastatic disease (mPFS and mOS of 29.7 months and 31.8 months, respectively). In previous clinical trials comparing immunotherapy combined with chemotherapy versus chemotherapy alone for stage IV esophageal cancer, PFS ranged from 5.8 to 7.3 months, and OS ranged from 12.6 to 17.2 months. Furthermore, the median duration of response (mDOR) for patients achieving CR/PR with first-line immunotherapy-chemotherapy combination but not receiving radiotherapy before disease progression was only 7.0 to 8.3 months (20, 44, 45). This indicates that adding radiotherapy consolidation after immunotherapy plus chemotherapy can prolong survival in stage IVb patients.

In the era of immunotherapy, patients with stage IVA disease can achieve enhanced control of local lesions and prolonged survival through combined immunotherapy and radiotherapy (42, 46). For patients with stage IVb disease, the addition of radiotherapy not only provides rapid relief of local symptoms such as dysphagia but also enables further control of local tumors after immunotherapy reduces tumor burden, thereby enhancing clinical benefit locally for stage IVb patients (20, 47, 48). In summary, the emergence of immunotherapy combined with chemotherapy as a first-line treatment has indeed provided patients with more therapeutic options and extended survival benefits. However, comparing OS rates reveals that adding radiotherapy as consolidation therapy after immunotherapy and chemotherapy significantly improves both OS and PFS in stage IV patients. Therefore, incorporating radiotherapy effectively improves the prognosis for patients with stage IV esophageal cancer.

## 4 The timing of adding consolidation radiotherapy

Previous studies have demonstrated that incorporating radiotherapy into the immunotherapy era can improve local control rates and OS in patients with stage IV esophageal cancer. However, the current challenge lies in determining the optimal timing for adding radiotherapy. Generally, there are three approaches to combining radiotherapy with immunotherapy: concurrent administration of radiotherapy and immunotherapy, or adding radiotherapy as consolidation therapy following immunotherapy.

If administered concurrently, the combination of two potent therapies may increase the risk of treatment-related adverse events. For patients with ulcerative disease, large tumor volume, thin esophageal walls, or invasion of major vessels (49, 50), adding concurrent radiotherapy during immunotherapy/immunotherapy-combined-chemotherapy may heighten the risk of treatment-related adverse events such as esophageal fistula, major vascular invasive bleeding, radiation pneumonitis, and bone marrow suppression. Esophageal fistula is a common and serious complication of esophageal cancer. Since both the esophagus and trachea develop from the embryonic foregut, they maintain close proximity in the superior mediastinum, separated by only 4 mm of connective tissue (51). Radiotherapy-induced local tissue injury, vascular disruption, and compromised mucosal barriers may exacerbate local inflammation following immune therapy activation of the body’s immune response, increasing susceptibility to tissue necrosis and fistula formation. Reports have indicated that the incidence of perforation significantly increases when immunotherapy is administered concurrently with esophageal radiotherapy (52). Additionally, in a study by Teng et al. (53) on overlapping lung toxicity from immunotherapy and radiotherapy, adding immunotherapy during radiotherapy was found to increase the probability of radiation pneumonitis. Furthermore, in a study by Liu et al. (54) on stage IV esophageal cancer, it was similarly found that concurrent chemoradiotherapy and immunotherapy significantly increased the incidence of radiation pneumonitis compared to radiotherapy alone (incidence rates of 10.8% vs 3.0%, respectively).The aforementioned EC-CRT-001 trial employed a regimen combining radiotherapy with concurrent immunotherapy and chemotherapy, which led to a higher incidence of treatment-related adverse events. Compared to previous clinical trials of radical concurrent chemoradiotherapy, EC-CRT-001 demonstrated a marked increase in Grade 1 and 2 pneumonia incidence (62% vs 7.3%) (42, 55). In summary, based on the comparison of the above data, the use of concurrent radiotherapy and immunotherapy combined with chemotherapy raises safety concerns. Therefore, to reduce the risk of treatment-related adverse events, a strategy of adding radiotherapy as consolidation therapy after immunotherapy may be considered.

As concluded by Hu et al. (43) mentioned above, radiotherapy is more suitable for administration after tumor burden reduction. Particularly for patients with stage IV esophageal cancer, who typically have a higher tumor burden, radiotherapy should be incorporated as consolidation therapy following immunotherapy and chemotherapy—approaches that are more effective at reducing systemic tumor burden. This raises the question: How long should the interval be between radiotherapy and immunotherapy? Wu et al. (56) compared the impact of different radiotherapy timing on the prognosis of esophageal cancer patients. Participants were divided into three groups: two groups received radiotherapy within 12 weeks before or after immunotherapy (Group A) and outside this 12-week window (Group B). The final results demonstrated significantly prolonged mPFS and mOS in Group A (7.43 months vs 2.76 months, 13.24 months vs 7.85 months, respectively), with comparable adverse event rates between groups (overall incidence: 85% vs 82%, with ≥Grade 3 adverse events at 12% vs 14%). This suggests that adding radiotherapy within 12 weeks after immunotherapy may further enhance treatment efficacy. For details regarding the immunotherapy-radiotherapy combination for ESCC mentioned above, please refer to **Table 1**. Although the study concluded that a 12-week interval yielded superior mOS and mPFS, current clinical trials for radiotherapy combined with immunotherapy require that the interval between standard radiotherapy and immunotherapy not exceed 8 weeks, while the interval between stereotactic body radiotherapy (SBRT) and immunotherapy is 1 week. The feasibility of this time interval can be addressed in studies of non-small cell lung cancer. For instance, a retrospective study by Bassanelli et al. (57) on stage IIIb/IV non-small cell lung cancer encompassing 95 patients and establishing an 8-week interval as the cutoff between immunotherapy and radiotherapy. The final results demonstrated significant benefits in mOS and mPFS for patients with an interval of 8 weeks or less compared to those with intervals exceeding 8 weeks (mOS: 22.4 months vs 8.6 months; mPFS: 8.7 months vs 6.3 months). Clinical trials for esophageal cancer also adhere to this time interval requirement. For instance, the ongoing NCT05183958 and NCT04512417. Both of these phase IV clinical trials for esophageal cancer employ an 8-week interval. These trials are currently ongoing, and relevant data have not yet been published.

**Table 1:** Summary of results from immunotherapy combined with radiotherapy for esophageal squamous cell carcinoma.

Study	Patient population	Treatment arms	Primary end point	Results	Ref.
EC-CRT-001 (Single-arm, phase 2 trial)	A total of 42 patients with ESCC (including 16 patients with stage IVa ESCC).	Patients received concurrent chemoradiotherapy consisting of five cycles of weekly intravenous paclitaxel (50 mg/m²) and cisplatin (25 mg/m²) during thoracic radiotherapy. Two cycles of toripalimab (240 mg) were administered intravenously on days 1 and 22, concurrent with the chemoradiotherapy. Radiotherapy was administered on 5 consecutive days per week with a target dose of 50.4 Gy in 28 fractions with intensity-modulated Radiotherapy.	The complete response rate at 3 months after completion of radiotherapy.	At 3 months after the completion of chemoradiotherapy, 26 (62%) of 42 patients had a complete response; nine (21%) patients had a partial response, two (5%) had stable disease, and five (12%) had progressive disease.	(42)
Hu et al. (Retrospective study)	A total of 72 patients with ESCC were included (42 cases were stage IVa and 30 cases were stage IVb).	Received first-line chemotherapy and immunotherapy with objective response (complete or partial remission, or stable disease), and prior to first disease progression also received radiotherapy to the primary lesion with a dose exceeding 40 Gy, with or without radiotherapy to metastatic lesions.	Adverse events and clinical outcomes.	Approximately 60% of patients developed CR or PR to initial chemoimmunotherapy. The mPFS and mOS were 13.5 months and 31.8 months, respectively.	(43)
NCT06478355 (Retrospective cohort study)	After propensity score matching, a total of 334 patients with ESCC were included (comprising 275 patients, including 59 stage IVa patients and stage IVb patients).	Received radiation therapy (ICRT) Group: Patients received platinum-based chemotherapy administered every 3 weeks for 4 to 6 cycles, primarily consisting of paclitaxel plus platinum or fluorouracil plus platinum. Anti-PD-1 antibodies, as systemic immunotherapy, primarily include sintilimab, camrelizumab, tislelizumab, and pembrolizumab. These are administered every 3 weeks until intolerable toxicity occurs, disease progression is observed, or for a duration of 1–2 years. Radiotherapy employs 6–8 MeV X-rays using volumetric modulated arc therapy (VMAT), intensity-modulated radiation therapy (IMRT), and tomotherapy (TOMO) techniques.	Adverse events and clinical outcomes.	After propensity score matching, 55.1% and 62.9% of patients in the ICRT and immunochemotherapy (ICT) groups, respectively, experienced death, with mOS of 34 months and 20 months, and mPFS of 16 months and 12 months.	(54)
Wu et al. (Retrospective study)	A total of 127 patients with ESCC (including 88 patients with stage IVb disease).	Radiotherapy is categorized into palliative radiotherapy, ablation therapy and conventional curative radiotherapy. Regarding the sequence of radiotherapy and immunotherapy, 8 patients received concurrent therapy with uninterrupted immunotherapy during radiotherapy, while the remaining 32 patients underwent sequential therapy with radiotherapy either before or after immunotherapy.	Alleviation of Dysphagia and Safety.	Dysphagia resolution was achieved in 64% (9/14) of patients in the RT group and 30% (8/27) in the No radiotherapy used (NRT) group.	(56)

In summary, the interval between immunotherapy and subsequent radiotherapy critically impacts both efficacy and safety: an interval exceeding 12 weeks leads to a significant decline in OS and PFS, suggesting diminished synergistic effects between radiotherapy and immunotherapy. Conversely, selecting an interval of 8 weeks or less may better prolong patient survival, though whether this represents the optimal solution requires confirmation through larger prospective clinical trials within the oncology community. Ultimately, determining the optimal time interval between immunotherapy and radiotherapy is essential for optimizing tumor treatment strategies, maximizing efficacy, and minimizing risks.

## 5 The definition of target volume and dose for consolidation radiotherapy

Under conventional radiotherapy, target volume definition primarily relies on anatomical imaging, with the gross tumor volume (GTV) encompassing both the primary tumor visible on imaging (GTVp) and metastatic lymph nodes (GTVnd). However, in the era of highly effective immunotherapy, is this traditional approach to target volume delineation still appropriate? In the early days of the immunotherapy era, discussions on improving radiotherapy target fields primarily focused on the choice between involved field irradiation (IFI) and extended neck irradiation (ENI). Ultimately, IFI emerged as the mainstream approach due to its superior ability to preserve lymph nodes in the tumor’s lymphatic drainage area and peripheral lymphocytes, thereby enhancing synergistic effects with immunotherapy (58–60). Subsequently, it was determined that target volumes should be delineated based on imaging studies following first-line systemic therapy (61). These perspectives collectively guide radiotherapy toward greater precision and minimalization. The aforementioned immunotherapy exerts its effects by infiltrating tumor tissues with lymphocytes (30). It is well known that lymphocytes are the most radiation-sensitive cells in the hematopoietic system and the entire body (62). A lethal dose of just 3 Gy is required to reduce the survival fraction of circulating lymphocytes by 90% (63), and lymphopenia is one of the most common adverse reactions to radiotherapy. In clinical practice, patients are often administered radiation doses sufficient to kill tumor cells. Consequently, some patients may experience significant bone marrow suppression during radiotherapy, leading to a substantial reduction in lymphocyte counts and a corresponding decrease in antitumor immune function (64). For example, in a study by Jing et al. (65) examining the impact of radiotherapy-induced severe lymphopenia (approximately grade 4) on the efficacy of immunotherapy in patients with non-small cell lung cancer, it was found that patients who developed severe lymphopenia experienced a significant reduction in mPFS compared to those who did not (17.1 months vs 37.7 months). Therefore, to meet the current demands for precision and minimalization in radiotherapy, we require more accurate diagnostic methods. Positron Emission Tomography (PET) imaging is now combined with Computed Tomography (CT), as PET cameras provide high sensitivity for tracer distribution while CT offers precise anatomical localization and attenuation correction. This approach, which tracks malignant tumors via the Warburg effect, has become the most sensitive and accurate diagnostic method (66, 67). Although positioning errors during radiotherapy are unavoidable, PET-CT-based techniques enable more precise delineation of the radiation target area. This maximizes the reduction of unnecessary tissue exposure, minimizes lymphocyte damage, and enhances the efficacy of immunotherapy.

The radiation dose varies depending on the treatment objectives for esophageal cancer patients. For example, neoadjuvant radiotherapy at 40–45 Gy is commonly administered to operable patients. For patients with unresectable locally advanced esophageal cancer, a dose of 50.4 Gy is typically used. If the patient’s general condition is favorable, a higher dose of 60 Gy may also be considered (68, 69). The answer can be found in the study by Tian et al. (70), which included 110 esophageal cancer patients, including 20 stage IV patients. Blood samples were collected at three time points: before radiotherapy, during radiotherapy, and one month after radiotherapy. A significant decrease in lymphocyte counts occurred during radiotherapy (90% of patients developed grade 3–4 lymphopenia, and 10% experienced Grade 1–2 neutropenia). The study also demonstrated negative correlations between the maximum heart dose, average heart dose, and average lung dose with the lowest lymphocyte count. This may be attributed to the high sensitivity of lymphocytes to radiation, where excessive doses can lead to lymphocyte death. Furthermore, the researchers identified the lowest lymphocyte count as an independent prognostic factor affecting patient outcomes (71). The impact of this lymphocyte depletion on immunotherapy is evident. As mentioned in the study by Jing et al. (65), immunotherapy—a treatment modality that relies on the body’s immune system to function—also experiences diminished efficacy due to reduced lymphocyte counts. Given these two reasons, the conventional fractionation doses for radiotherapy require further reassessment in the era of immunotherapy.

Clinically, with continuous advancements in radiotherapy techniques, treatment modalities have become increasingly diverse. For instance, Lugade et al. (72) observed in radiotherapy for B16 melanoma that a single 15Gy fraction significantly increased antigen-presenting cells and T cell counts in tumor-draining lymph nodes compared to high-dose hyperfractionated radiotherapy at 5Gy/3 fractions. Concurrently, preclinical studies indicate that low-dose radiotherapy also influences the tumor microenvironment. For instance, Klug et al. (73) demonstrated that a regimen of 0.5 Gy × 4 fractions effectively reprograms tumor-infiltrating myeloid cells, promoting the recruitment of cytotoxic T lymphocytes (CTLs). Regarding clinical trials combining low-dose radiotherapy with immunotherapy, only NCT03061162 has published results, reporting a CR of 95.8%, meaning nearly all patients achieved remission or stabilization. This trial remains under follow-up, and we anticipate encouraging outcomes in the near future (74). Additionally, ongoing clinical trials combining low-dose radiotherapy with immunotherapy include NCT02444741, NCT02710253, NCT04622228, and others.

In summary, the lowest lymphocyte count is an independent prognostic factor for patients. Compared to conventional fractionation radiotherapy, both high-fractionation radiotherapy and low-dose radiotherapy demonstrate significantly superior lymphocyte infiltration in the tumor microenvironment. Therefore, enhancing the efficacy of radiotherapy combined with immunotherapy cannot be achieved solely by increasing the conventional radiotherapy dose; alternative fractionation methods should be considered. The above theory provides a foundation for how hypofractionated radiotherapy and low-dose radiotherapy enhance the efficacy of combined immunotherapy and radiotherapy. Although several clinical trials have yielded conclusions, the specific fractionation schemes and doses still require large-scale clinical trials for further clarification.

## 6 Conclusion

Based on prior preclinical studies and clinical trials, combining immunotherapy with radiotherapy demonstrates synergistic effects. Given the significant therapeutic benefit observed when adding immunotherapy to radical chemoradiotherapy in patients with inoperable esophageal cancer, we hypothesize that incorporating radiotherapy as a local consolidation modality following chemotherapy plus immunotherapy in stage IV esophageal cancer patients is safe and feasible. However, clinical research in this area remains scarce. Key aspects such as the efficacy of this regimen, the optimal timing for combining immunotherapy and radiotherapy, and the appropriate radiation target fields and doses remain unknown. With ongoing advancements in relevant drugs and the expansion of clinical trials, we believe this treatment approach will be better utilized in the near future, and these questions will be resolved.

## References

[1] Siegel RL Miller KD Fuchs HE Jemal A . Cancer statistics, 2022. CA Cancer J Clin. (2022) 72:7–33. doi: 10.3322/caac.21708 35020204

[2] Yang H Wang F Hallemeier CL Lerut T Fu J . Oesophageal cancer. Lancet. (2024) 404:1991–2005. doi: 10.1016/s0140-6736(24)02226-8 39550174

[3] Zhu H Ma X Ye T Wang H Wang Z Liu Q . Esophageal cancer in China: Practice and research in the new era. Int J Cancer. (2023) 152:1741–51. doi: 10.1002/ijc.34301 36151861

[4] Cao W Qin K Li F Chen W . Comparative study of cancer profiles between 2020 and 2022 using global cancer statistics (GLOBOCAN). J Natl Cancer Cent. (2024) 4:128–34. doi: 10.1016/j.jncc.2024.05.001 39282581 PMC11390618

[5] Morgan E Soerjomataram I Rumgay H Coleman HG Thrift AP Vignat J . The global landscape of esophageal squamous cell carcinoma and esophageal adenocarcinoma incidence and mortality in 2020 and projections to 2040: New estimates from GLOBOCAN 2020. Gastroenterology. (2022) 163:649–658.e642. doi: 10.1053/j.gastro.2022.05.054 35671803

[6] Veugelers PJ Porter GA Guernsey DL Casson AG . Obesity and lifestyle risk factors for gastroesophageal reflux disease, Barrett esophagus and esophageal adenocarcinoma. Dis Esophagus. (2006) 19:321–8. doi: 10.1111/j.1442-2050.2006.00602.x 16984526

[7] Saad AM Al-Husseini MJ Elgebaly A Aboshady OA Salahia S Abdel-Rahman O . Impact of prior Malignancy on outcomes of stage IV esophageal carcinoma: SEER based study. Expert Rev Gastroenterol Hepatol. (2018) 12:417–23. doi: 10.1080/17474124.2018.1426458 29316808

[8] Hu K Kang N Liu Y Guo D Jing W Lu J . Proposed revision of N categories to the 8th edition of the AJCC-TNM staging system for non-surgical esophageal squamous cell cancer. Cancer Sci. (2019) 110:717–25. doi: 10.1111/cas.13891 30467921 PMC6361553

[9] Shah MA Kennedy EB Alarcon-Rozas AE Alcindor T Bartley AN Malowany AB . Immunotherapy and targeted therapy for advanced gastroesophageal cancer: ASCO guideline. J Clin Oncol. (2023) 41:1470–91. doi: 10.1200/jco.22.02331 36603169

[10] Jiang W Zhang B Xu J Xue L Wang L . Current status and perspectives of esophageal cancer: a comprehensive review. Cancer Commun (Lond). (2025) 45:281–331. doi: 10.1002/cac2.12645 39723635 PMC11947622

[11] Ajani JA D'Amico TA Bentrem DJ Cooke D Corvera C Das P . Esophageal and esophagogastric junction cancers, version 2.2023, NCCN clinical practice guidelines in oncology. J Natl Compr Canc Netw. (2023) 21:393–422. doi: 10.6004/jnccn.2023.0019 37015332

[12] Chen Y Ye J Zhu Z Zhao W Zhou J Wu C . Comparing paclitaxel plus fluorouracil versus cisplatin plus fluorouracil in chemoradiotherapy for locally advanced esophageal squamous cell cancer: a randomized, multicenter, phase III clinical trial. J Clin Oncol. (2019) 37:1695–703. doi: 10.1200/jco.18.02122 30920880 PMC6638596

[13] Bleiberg H Conroy T Paillot B Lacave AJ Blijham G Jacob JH . Randomised phase II study of cisplatin and 5-fluorouracil (5-FU) versus cisplatin alone in advanced squamous cell oesophageal cancer. Eur J Cancer. (1997) 33:1216–20. doi: 10.1016/s0959-8049(97)00088-9 9301445

[14] Cooper JS Guo MD Herskovic A Macdonald JS Martenson JA Jr Al-Sarraf M . Chemoradiotherapy of locally advanced esophageal cancer: long-term follow-up of a prospective randomized trial (RTOG 85-01). Radiation Therapy Oncology Group. Jama. (1999) 281:1623–7. doi: 10.1001/jama.281.17.1623 10235156

[15] Muro K Hamaguchi T Ohtsu A Boku N Chin K Hyodo I . A phase II study of single-agent docetaxel in patients with metastatic esophageal cancer. Ann Oncol. (2004) 15:955–9. doi: 10.1093/annonc/mdh231 15151954

[16] Lyu J Li T Wang Q Li F Diao P Liu L . Outcomes of concurrent chemoradiotherapy versus chemotherapy alone for stage IV esophageal squamous cell carcinoma: a retrospective controlled study. Radiat Oncol. (2018) 13:233. doi: 10.1186/s13014-018-1183-y 30477531 PMC6257959

[17] Killock D . ICI for resected stage IV melanoma. Nat Rev Clin Oncol. (2020) 17:450. doi: 10.1038/s41571-020-0397-8 32472086

[18] Cascone T Fradette J Pradhan M Gibbons DL . Tumor immunology and immunotherapy of non-small-cell lung cancer. Cold Spring Harb Perspect Med. (2022) 12(2022):a037895. doi: 10.1101/cshperspect.a037895 34580079 PMC8957639

[19] Li N Sohal D . Current state of the art: immunotherapy in esophageal cancer and gastroesophageal junction cancer. Cancer Immunol Immunother. (2023) 72:3939–52. doi: 10.1007/s00262-023-03566-5 37995002 PMC10991203

[20] Sun JM Shen L Shah MA Enzinger P Adenis A Doi T . Pembrolizumab plus chemotherapy versus chemotherapy alone for first-line treatment of advanced oesophageal cancer (KEYNOTE-590): a randomised, placebo-controlled, phase 3 study. Lancet. (2021) 398:759–71. doi: 10.1016/s0140-6736(21)01234-4 34454674

[21] Luo H Lu J Bai Y Mao T Wang J Fan Q . Effect of camrelizumab vs placebo added to chemotherapy on survival and progression-free survival in patients with advanced or metastatic esophageal squamous cell carcinoma: The ESCORT-1st randomized clinical trial. Jama. (2021) 326:916–25. doi: 10.1001/jama.2021.12836 34519801 PMC8441593

[22] Wu J Qin Y Li K Hao W Liu X Xing W . Recurrence patterns and long-term survival of locally advanced esophageal cancer patients with pathological complete response after different neoadjuvant therapies followed by surgery. BMC Cancer. (2025) 25:1135. doi: 10.1186/s12885-025-14548-4 40597852 PMC12220425

[23] Arina A Gutiontov SI Weichselbaum RR . Radiotherapy and immunotherapy for cancer: From “systemic” to “multisite. Clin Cancer Res. (2020) 26:2777–82. doi: 10.1158/1078-0432.CCR-19-2034 PMC10759929 32047000

[24] Zhou S Yang H . Radiotherapy modulates autophagy to reshape the tumor immune microenvironment to enhance anti-tumor immunity in esophageal cancer. Biochim Biophys Acta Rev Cancer. (2025) 1880:189302. doi: 10.1016/j.bbcan.2025.189302 40120778

[25] Ngwa W Irabor OC Schoenfeld JD Hesser J Demaria S Formenti SC . Using immunotherapy to boost the abscopal effect. Nat Rev Cancer. (2018) 18:313–22. doi: 10.1038/nrc.2018.6 29449659 PMC5912991

[26] Wang D Zhang X Gao Y Cui X Yang Y Mao W . Research progress and existing problems for abscopal effect. Cancer Manag Res. (2020) 12:6695–706. doi: 10.4324/9781351215503-2 PMC7413699 32801902

[27] Yin Z Li C Wang J Xue L . Myeloid-derived suppressor cells: Roles in the tumor microenvironment and tumor radiotherapy. Int J Cancer. (2019) 144:933–46. doi: 10.1002/ijc.31744 29992569

[28] Tagliaferri L Lancellotta V Fionda B Mangoni M Casà C Di Stefani A . Immunotherapy and radiotherapy in melanoma: a multidisciplinary comprehensive review. Hum Vaccin Immunother. (2022) 18:1903827. doi: 10.1080/21645515.2021.1903827 33847208 PMC9122308

[29] Zhai D An D Wan C Yang K . Radiotherapy: Brightness and darkness in the era of immunotherapy. Transl Oncol. (2022) 19:101366. doi: 10.1016/j.tranon.2022.101366 35219093 PMC8881489

[30] Wen L Tong F Zhang R Chen L Huang Y Dong X . The research progress of PD-1/PD-L1 inhibitors enhancing radiotherapy efficacy. Front Oncol. (2021) 11:799957. doi: 10.3389/fonc.2021.799957 34956911 PMC8695847

[31] Ko EC Raben D Formenti SC . The integration of radiotherapy with immunotherapy for the treatment of non-small cell lung cancer. Clin Cancer Res. (2018) 24:5792–806. doi: 10.1158/1078-0432.ccr-17-3620 29945993

[32] Chakraborty M Abrams SI Camphausen K Liu K Scott T Coleman CN . Irradiation of tumor cells up-regulates Fas and enhances CTL lytic activity and CTL adoptive immunotherapy. J Immunol. (2003) 170:6338–47. doi: 10.4049/jimmunol.170.12.6338 12794167

[33] Reits EA Hodge JW Herberts CA Groothuis TA Chakraborty M Wansley EK . Radiation modulates the peptide repertoire, enhances MHC class I expression, and induces successful antitumor immunotherapy. J Exp Med. (2006) 203:1259–71. doi: 10.1083/jcb1734oia6 PMC3212727 16636135

[34] Pitroda SP Chmura SJ Weichselbaum RR . Integration of radiotherapy and immunotherapy for treatment of oligometastases. Lancet Oncol. (2019) 20:e434–42. doi: 10.1016/s1470-2045(19)30157-3 31364595

[35] Mondini M Levy A Meziani L Milliat F Deutsch E . Radiotherapy-immunotherapy combinations - perspectives and challenges. Mol Oncol. (2020) 14:1529–37. doi: 10.1002/1878-0261.12658 32112478 PMC7332212

[36] Chen Y Gao M Huang Z Yu J Meng X . SBRT combined with PD-1/PD-L1 inhibitors in NSCLC treatment: a focus on the mechanisms, advances, and future challenges. J Hematol Oncol. (2020) 13:105. doi: 10.1186/s13045-020-00940-z 32723363 PMC7390199

[37] Rodríguez-Ruiz ME Vanpouille-Box C Melero I Formenti SC Demaria S . Immunological mechanisms responsible for radiation-induced abscopal effect. Trends Immunol. (2018) 39:644–55. doi: 10.1016/j.it.2018.06.001 30001871 PMC6326574

[38] van Hagen P Hulshof MC van Lanschot JJ Steyerberg EW van Berge Henegouwen MI Wijnhoven BP . Preoperative chemoradiotherapy for esophageal or junctional cancer. N Engl J Med. (2012) 366:2074–84. doi: 10.1056/nejmoa1112088 22646630

[39] Kawamoto T Nihei K Sasai K Karasawa K . Palliative radiotherapy and chemoradiotherapy in stage IVA/B esophageal cancer patients with dysphagia. Int J Clin Oncol. (2018) 23:1076–83. doi: 10.1007/s10147-018-1324-1 30066207

[40] Akl FM Elsayed-Abd-Alkhalek S Salah T . Palliative concurrent chemoradiotherapy in locally advanced and metastatic esophageal cancer patients with dysphagia. Ann Palliat Med. (2013) 2:118–23. doi: 10.3978/j.issn.2224-5820.2013.05.01 25842093

[41] Ikeda E Kojima T Kaneko K Minashi K Onozawa M Nihei K . Efficacy of concurrent chemoradiotherapy as a palliative treatment in stage IVB esophageal cancer patients with dysphagia. Jpn J Clin Oncol. (2011) 41:964–72. doi: 10.1093/jjco/hyr088 21742654

[42] Zhu Y Wen J Li Q Chen B Zhao L Liu S . Toripalimab combined with definitive chemoradiotherapy in locally advanced oesophageal squamous cell carcinoma (EC-CRT-001): a single-arm, phase 2 trial. Lancet Oncol. (2023) 24:371–82. doi: 10.1016/s1470-2045(23)00060-8 36990609

[43] Hu H-H Xu X Li X-Y Zeng Y Li Y Song X-Y . The value of intervention with radiotherapy after first-line chemo-immunotherapy in locally advanced or metastatic esophageal squamous cell carcinoma: A multi-center retrospective study. Clin Transl Radiat Oncol. (2024) 48:100818. doi: 10.1016/j.ctro.2024.100818 39091465 PMC11292253

[44] Doki Y Ajani JA Kato K Xu J Wyrwicz L Motoyama S . Nivolumab combination therapy in advanced esophageal squamous-cell carcinoma. N Engl J Med. (2022) 386:449–62. doi: 10.1056/nejmoa2111380 35108470

[45] Xu J Kato K Raymond E Hubner RA Shu Y Pan Y . Tislelizumab plus chemotherapy versus placebo plus chemotherapy as first-line treatment for advanced or metastatic oesophageal squamous cell carcinoma (RATIONALE-306): a global, randomised, placebo-controlled, phase 3 study. Lancet Oncol. (2023) 24:483–95. doi: 10.1016/s1470-2045(23)00108-0 37080222

[46] Wang R Ling Y Chen B Zhu Y Hu Y Liu M . Long-term survival and post-hoc analysis of toripalimab plus definitive chemoradiotherapy for oesophageal squamous cell carcinoma: insights from the EC-CRT-001 phase II trial. EClinicalMedicine. (2024) 75:102806. doi: 10.1016/j.eclinm.2024.102806 39281099 PMC11402426

[47] Zhang Y Dong S Xu R Yang Y Zheng Z Wang X . Prognostic and predictive role of COX-2, XRCC1 and RASSF1 expression in patients with esophageal squamous cell carcinoma receiving radiotherapy. Oncol Lett. (2017) 13:2549–56. doi: 10.3892/ol.2017.5780 28454432 PMC5403488

[48] Kato K Sun JM Shah MA Enzinger PC Adenis A Doi T . LBA8_PR Pembrolizumab plus chemotherapy versus chemotherapy as first-line therapy in patients with advanced esophageal cancer: The phase 3 KEYNOTE-590 study. Ann Oncol. (2020) 31:S1192–3. doi: 10.1016/j.annonc.2020.08.2298 38826717

[49] Wu L Wang C Tan X Cheng Z Zhao K Yan L . Radiomics approach for preoperative identification of stages I-II and III-IV of esophageal cancer. Chin J Cancer Res. (2018) 30:396–405. doi: 10.21147/j.issn.1000-9604.2018.04.02 30210219 PMC6129566

[50] Xin Z Liu Q Ai D Chen K Mariamidze E Sumon MA . Radiotherapy for advanced esophageal cancer: from palliation to curation. Curr Treat Options Oncol. (2023) 24:1568–79. doi: 10.1007/s11864-023-01134-8 37812321

[51] Shamji FM Inculet R . Management of Malignant tracheoesophageal fistula. Thorac Surg Clin. (2018) 28:393–402. doi: 10.1016/j.thorsurg.2018.04.007 30054077

[52] Peng F Bao Y Lian HM Niu SQ . Increased esophageal perforation after combination of anti-PD-1 immunotherapy with radiotherapy for esophageal carcinoma. Int J Radiat Oncol Biol Phys. (2021) 111:S102. doi: 10.1016/j.ijrobp.2021.07.237 38826717

[53] Teng F Li M Yu J . Radiation recall pneumonitis induced by PD-1/PD-L1 blockades: mechanisms and therapeutic implications. BMC Med. (2020) 18:275. doi: 10.1186/s12916-020-01718-3 32943072 PMC7499987

[54] Liu X Wen J Zhang Y Wang C Liu Y Qian P . Impact of first-line chemoimmunotherapy with or without radiotherapy on the prognosis of patients with locally advanced or metastatic esophageal squamous cell carcinoma: a multicenter, real-world, retrospective cohort study from China (NCT06478355). Front Immunol. (2025) 16:1633930. doi: 10.3389/fimmu.2025.1633930 40791599 PMC12336177

[55] Zhan PL Canavan ME Ermer T Pichert MD Li AX Maduka RC . Utilization and outcomes of radiation in stage IV esophageal cancer. JTO Clin Res Rep. (2022) 3:100429. doi: 10.1016/j.jtocrr.2022.100429 36483656 PMC9722471

[56] Wu X Li Y Zhang K Guo Z Li Y Zhao F . Immunotherapy with or without radiotherapy for metastatic or recurrent esophageal squamous cell carcinoma: a real-world study. Clin Transl Radiat Oncol. (2023) 38:130–7. doi: 10.1016/j.ctro.2022.10.011 36425536 PMC9678957

[57] Bassanelli M Ricciuti B Giannarelli D Cecere FL Roberto M Giacinti S . Systemic effect of radiotherapy before or after nivolumab in lung cancer: an observational, retrospective, multicenter study. Tumori. (2022) 108:250–7. doi: 10.1177/03008916211004733 33818208

[58] Marciscano AE Ghasemzadeh A Nirschl TR Theodros D Kochel CM Francica BJ . Elective nodal irradiation attenuates the combinatorial efficacy of stereotactic radiation therapy and immunotherapy. Clin Cancer Res. (2018) 24:5058–71. doi: 10.1158/1078-0432.ccr-17-3427 29898992 PMC6532976

[59] Darragh LB Gadwa J Pham TT Van Court B Neupert B Olimpo NA . Elective nodal irradiation mitigates local and systemic immunity generated by combination radiation and immunotherapy in head and neck tumors. Nat Commun. (2022) 13:7015. doi: 10.1038/s41467-022-34676-w 36385142 PMC9668826

[60] Zheng J Zheng Z Zhang T Chen X Pang Q Wang P . Optimization of radiation target volume for locally advanced esophageal cancer in the immunotherapy era. Expert Opin Biol Ther. (2024) 24:1221–32. doi: 10.1080/14712598.2024.2423009 39460561

[61] Giraud P Lacornerie T Mornex F . Radiothérapie des cancers primitifs du poumon. Cancer/Radiothérapie. (2016) 20:S147–56. doi: 10.1684/ito.2016.0034 27516052

[62] Kutlaca R Seshadri R Morley AA . Sensitivity of lymphocytes from patients with cancer to x- and ultraviolet radiation. Cancer. (1984) 54:2952–5. doi: 10.1002/1097-0142(19841215)54:12<2952::aid-cncr2820541223>3.0.co;2-w 6498769

[63] Nakamura N Kusunoki Y Akiyama M . Radiosensitivity of CD4 or CD8 positive human T-lymphocytes by an *in vitro* colony formation assay. Radiat Res. (1990) 123:224–7. doi: 10.2307/3577549 2117766

[64] Cortiula F Reymen B Peters S Van Mol P Wauters E Vansteenkiste J . Immunotherapy in unresectable stage III non-small-cell lung cancer: state of the art and novel therapeutic approaches. Ann Oncol. (2022) 33:893–908. doi: 10.1016/j.annonc.2022.06.013 35777706

[65] Jing W Xu T Wu L Lopez PB Grassberger C Ellsworth SG . Severe radiation-induced lymphopenia attenuates the benefit of durvalumab after concurrent chemoradiotherapy for NSCLC. JTO Clin Res Rep. (2022) 3:100391. doi: 10.1016/j.jtocrr.2022.100391 36089921 PMC9449658

[66] Basu S Hess S Nielsen Braad PE Olsen BB Inglev S Høilund-Carlsen PF . The basic principles of FDG-PET/CT imaging. Pet Clin. (2014) 9:355–70. doi: 10.1016/j.cpet.2014.07.006 26050942

[67] Kröger K Pepper NB Ventura D Troschel FM Backhaus P Rahbar K . FAPI-PET/CT guided radiotherapy for patients with esophageal cancer. Radiat Oncol. (2025) 20:29. doi: 10.1186/s13014-025-02606-x 40022163 PMC11871644

[68] Lin SH Hobbs BP Verma V Tidwell RS Smith GL Lei X . Randomized phase IIB trial of proton beam therapy versus intensity-modulated radiation therapy for locally advanced esophageal cancer. J Clin Oncol. (2020) 38:1569–79. doi: 10.1200/jco.19.02503 32160096 PMC7213588

[69] Lewis S Lukovic J . Neoadjuvant therapy in esophageal cancer. Thorac Surg Clin. (2022) 32:447–56. doi: 10.1016/j.thorsurg.2022.06.003 36266032

[70] Tang C Liao Z Gomez D Levy L Zhuang Y Gebremichael RA . Lymphopenia association with gross tumor volume and lung V5 and its effects on non-small cell lung cancer patient outcomes. Int J Radiat Oncol Biol Phys. (2014) 89:1084–91. doi: 10.1016/j.ijrobp.2014.04.025 25035212

[71] Tian X Hou Y Guo J Wu H Nie L Wang H . Effect of intensity modulated radiotherapy on lymphocytes in patients with esophageal squamous cell carcinoma and its clinical significance. Front Oncol. (2023) 13:1096386. doi: 10.3389/fonc.2023.1096386 36959779 PMC10028288

[72] Lugade AA Moran JP Gerber SA Rose RC Frelinger JG Lord EM . Local radiation therapy of B16 melanoma tumors increases the generation of tumor antigen-specific effector cells that traffic to the tumor. J Immunol. (2005) 174:7516–23. doi: 10.4049/jimmunol.174.12.7516 15944250

[73] Klug F Prakash H Huber PE Seibel T Bender N Halama N . Low-dose irradiation programs macrophage differentiation to an iNOS^+^/M1 phenotype that orchestrates effective T cell immunotherapy. Cancer Cell. (2013) 24:589–602. doi: 10.1016/j.ccr.2013.09.014 24209604

[74] Yang Y Yan J Liu J Gao S Du J Wei J . Phase 2 study of pulsed low dose rate radiation therapy for gastric cancer patients with peritoneal metastasis. Int J Radiat Oncol Biol Phys. (2017) 99:E201. doi: 10.1016/j.ijrobp.2017.06.1083 38826717

